# An aqueous electrolyte of the widest potential window and its superior capability for capacitors

**DOI:** 10.1038/srep45048

**Published:** 2017-03-21

**Authors:** Hiroshi Tomiyasu, Hirokazu Shikata, Koichiro Takao, Noriko Asanuma, Seiichi Taruta, Yoon-Yul Park

**Affiliations:** 1 Qualtec Co. Ltd., 4-230 Sambo-cho, Sakai, Osaka, 590-0906, Japan; 2 Laboratory for Advanced Nuclear Energy, Institute of Innovative Research, Tokyo Institute of Technology O-okayama, Meguro-ku, Tokyo 152-8550, Japan; 3 School of Engineering, Tokai University, Kitakaname, Hiratsuka 259-1292, Japan; 4 Faculty of Engineering, Shinshu University, 4-17-1, Wakasato 380-8553, Japan

## Abstract

A saturated aqueous solution of sodium perchlorate (SSPAS) was found to be electrochemically superior, because the potential window is remarkably wide to be approximately 3.2 V in terms of a cyclic voltammetry. Such a wide potential window has never been reported in any aqueous solutions, and this finding would be of historical significance for aqueous electrolyte to overcome its weak point that the potential window is narrow. In proof of this fact, the capability of SSPAS was examined for the electrolyte of capacitors. Galvanostatic charge-discharge measurements showed that a graphite-based capacitor containing SSPAS as an electrolyte was stable within 5% deviation for the 10,000 times repetition at the operating voltage of 3.2 V without generating any gas. The SSPAS worked also as a functional electrolyte in the presence of an activated carbon and metal oxides in order to increase an energy density. Indeed, in an asymmetric capacitor containing MnO_2_ and Fe_3_O_4_ mixtures in the positive and negative electrodes, respectively, the energy density enlarged to be 36.3 Whkg^−1^, which belongs to the largest value in capacitors. Similar electrochemical behaviour was also confirmed in saturated aqueous solutions of other alkali and alkaline earth metal perchlorate salts.

It has been generally understood that aqueous electrolytes have many advantages compared with non-aqueous solvents with respect to electrochemical behaviour as well as economic and environmental impacts. However, there exists serious disadvantage in aqueous electrolytes that water is easily electrolyzed to generate gases. This is attributed to the narrow electrochemical potential windows of aqueous solutions. As a matter of fact the thermodynamic potential window of water is known to be 1.23 V. For the study on capacitors the width of potential window is essentially important, since the energy storage in electric double-layer capacitors is proportional to the square of applied voltage^1^. Therefore, the use of aqueous electrolytes in capacitors should be accompanied by the extension of the potential window. Numerous attempts have been reported to extend the potential window of water. In 5 M (M = mol dm^−3^) LiNO_3_ aqueous solutions^2^, the potential windows were determined to be 2.3 V(−0.55~1.75 V) by a constant current (50 μA cm^−2^) method. The potential window of 2.0 V was reported in a 0.1 M KCl unbuffered aqueous electrolyte by using nanostructured platinum electrodes, where the change in local acidity at the electrodes contributed to the expansion of the potential window. A gel electrolyte^3^ consisting of the mixture of polyvinyl alcohol and KOH aqueous solution gave the potential window of 2.0 V. Much attention has been focused on electrode materials of high overpotentials for oxygen and/or hydrogen evolution. An attempt was made for supercapacitors (we will call just capacitor hereafter) consisting of the composites of carbon nanotubes with MnO_2_ in the positive electrode/active carbon in the negative electrode in KNO_3_ aqueous solution gave 2.0 V for the maximum charging voltage^4^. Other attempts to improve the operation voltage of aqueous electrolytes were reported by using asymmetric and symmetric capacitors, i.e. MnO_2_/carbon asymmetric capacitors^5^,^6^,^7^,^8^, and a carbon/carbon symmetric capacitor^9^. In the case of MnO_2_/graphene capacitor in 1 M Na_2_SO_4_ aqueous solution^5^, a cell voltage was 2.0 V giving a large energy density of 25.2 Whkg^−1^. In asymmetric aqueous capacitors, MnO_2_/nanoporous activated carbon^6^ and MnO_2_/high purity carbon nanotubes^7^, the operation voltages were 1.5 V, and 2 V, respectively. In a symmetric carbon/carbon capacitor consisting of a homogeneous mixture of 80% activated carbon, 10% of acetylene black and 10% of binder^9^, the operation voltage was 1.6 V. In a review paper^1^, data are summarized on a variety of capacitors using various aqueous electrolytes and various electrodes. However, the potential window listed was 1.8 V at the maximum.

As a result from earlier studies^1^,^2^,^3^,^4^,^5^,^6^,^7^,^8^,^9^,^10^, the potential window of aqueous electrolytes is limited at around 2 V even though by using any specific electrode materials. Although the expansion of the water window to 2 V is a great advance in aqueous electrolytes, it is still not enough to replace non-aqueous electrolytes with aqueous electrolytes.

The aim of the present study is to expand the potential window of aqueous electrolytes based on the view point of solution chemistry. At first, a question arises why water is easily electrolyzed. There are two major impacts to contribute the electrolysis of water. One is the acid equilibrium of water and the other is the hydrogen bond between water molecules. It is well known that electrolysis of water is sensitive in the acidity. However, to our knowledge, no one has paid attention electrochemically with respect to the hydrogen bond of water. The hydrogen bond is originated between a hydrogen and a neighboring oxygen (or other electronegative atoms) at the distance shorter than 0.3 nm. There is a trade-off relationship between the covalent O-H bond and the hydrogen bond^11^, that is, weaker the hydrogen bond, stronger the O-H bond. This leads to a conclusion that the O-H bond of water should be strengthened if a water molecule is isolated from others because of the weakened hydrogen bond. As a matter of fact, the O-H bond strength in isolated gaseous water is even stronger than the O-H bonds of methanol and ethanol, i.e. 497 kJmol^−1^ in water is compared with 436 and 437 kJmol^−1^ of methanol and ethanol^12^. The addition of ions into water disturbs the hydrogen bond network, where the electronegative oxygen atom of water is attracted to positive ions and the electron poor hydrogen atom is attracted to negative ions.

In the case of saturated sodium perchlorate aqueous solution (SSPAS), since sodium perchlorate is extremely soluble in water (the solubility is 219.6 g in 100 g water at 25 °C), only 3.3 water molecules exist per one sodium perchlorate molecule. Under such a condition the hydrogen bond could be destroyed almost completely due to the lack of neighboring water molecules and also the strong hydration to Na^+^ and ClO_4_^−^. The acid equilibrium could be also limited under the condition that the content of free water is too low for H^+^ to form hydronium ion, H_3_O^+^. Note that H^+^ has considerably large hydration energy among mono-valent ions, and hence only the hydrated species is able to exist in aqueous solutions.

We do not know in detail, but it is true that the evaporation rate of SSPAS is extremely slow owing to the strong hydration. This unusual phenomenon is known to be a theoretical base that water may exist in Mars^13^. Similar behaviour was also reported in a saturated magnesium perchlorate aqueous solution^13^. This suggests that the saturated magnesium perchlorate aqueous solution (SMPAS) would possibly be a superior electrolyte. Consequently, it is not surprising that the potential windows of SSPAS is extended to the region of non-aqueous solvents because of the enhanced strength of O-H bond due to the loss of hydrogen bond.

In the present paper, we will report a definite evidence for the widest potential window of SSPAS and demonstrate its superior capability as the electrolyte for capacitors using only low cost standard materials for this purpose.

## Results

### Cyclic voltammetry

**Figure 1:**
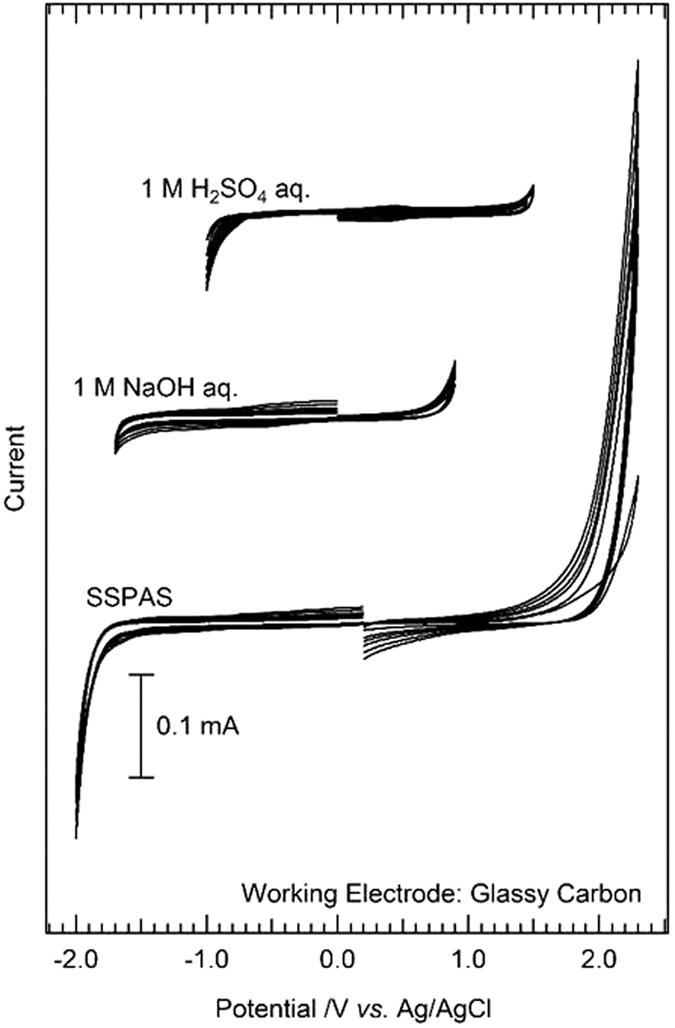
Cyclic voltammogram of SSPAS and those of 1 M H_2_SO_4_ and 1 M NaOH aqueous solutions using a glassy carbon for a working electrode. Measurements were made separately for the positive scan to 2.3 V and for the negative scan to −2.0 V at the scan rate from 30 mVs^−1^ to 200 mVs^−1^ at 25 °C.

Cyclic voltammetry (CV) is most commonly used to investigate the electrochemical properties of electrolytes and electrode materials^14^,^15^,^16^,^17^,^18^,^19^. The CV measurements for SSPAS were performed together with typical acidic (1 M H_2_SO_4_) and basic (1 M NaOH) aqueous solutions by a BAS CV-50W using a glassy carbon for a working electrode, a platinum counter electrode and an Ag/AgCl reference electrode under the argon atmosphere. Measurements were made separately for the positive scan to 2.3 V and for the negative scan to −2.0 V at the scan rate from 30 mVs^−1^ to 200 mVs^−1^ at 25 °C. The cyclic voltammograms are exhibited in Fig. 1. Because of large over potential of the glassy carbon used for the working electrode, the potential windows are relatively wide. Even though, the potential window of SSPAS is remarkably large compared with those of typical acidic and basic solutions. The potential window of SSPAS was determined to be approximately 3.2 V from the cyclic voltammogram.

### Capability of SSPAS as an electrolyte in graphite-based capacitors

**Figure 2:**
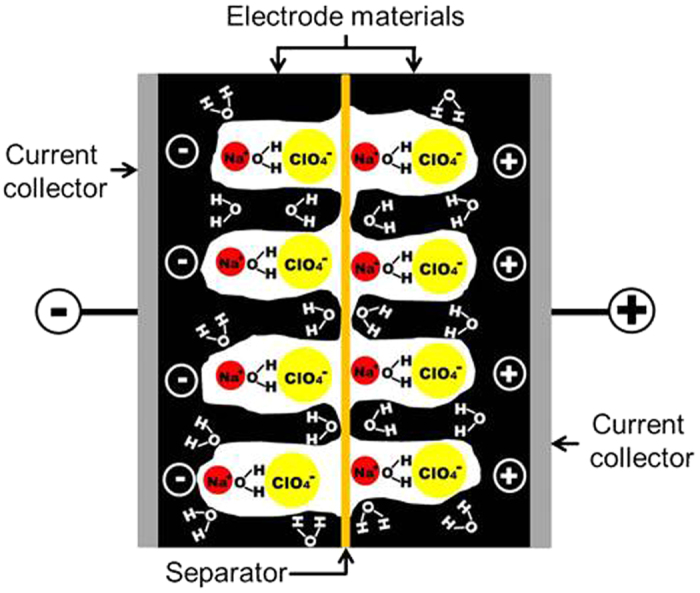
Estimated structure of a capacitor using SSPAS as an electrolyte. At least one water molecule should be strongly hydrated to NaClO_4_^13^ and number of free water molecules would not be enough to form a hydrogen bond.

A proposed scheme of the electrical double-layer capacitor for SSPAS is illustrated in Fig. 2 under the charging, where Na^+^ and ClO_4_^−^ might be strongly attracted to negative and positive electrodes respectively because of the weakened shield due to limited number of water molecules.

**Figure 3:**
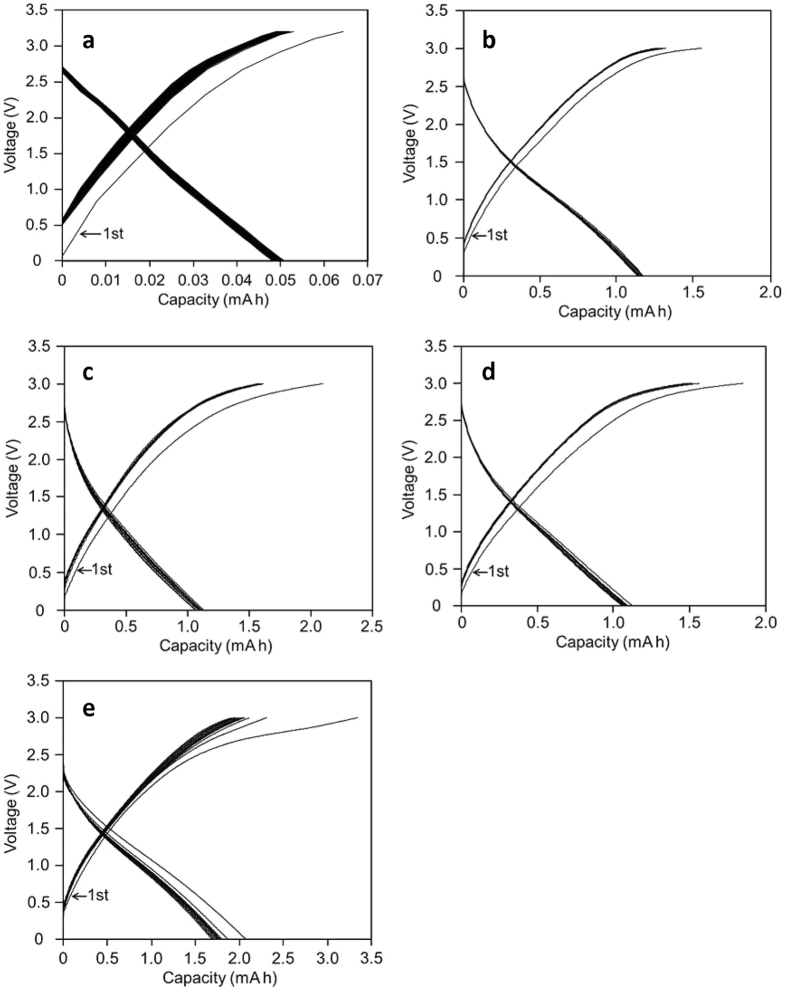
Galvanostatic charge-discharge cycles for various graphite-based symmetric capacitors. (**a**) Charge and discharge cycles of a symmetric graphite-based capacitor with a CEM as the separator for10,000 times repetition; (**b**) Charge and discharge cycles of a symmetric graphite-based capacitor containing 20% of AC with a CEM, (**c**) with a MF and (**d**) with a FP as the separator (**e**). Charge and discharge cycles of a symmetric graphite capacitor containing 40% of AC with a MF as the separator. Other specific conditions are described in Table 1.

The capability of SSPAS as the electrolyte for capacitors was examined first by a simple symmetrical graphite-based capacitor consisting of the following mixture: 80% graphite, 10% acetylene black and 10% carbon felt (we call hereafter a graphite acetylene black mixture or simply a graphite mixture abbreviated as GA). Then GA was made to form a thin film under the pressure as described later, where SSPAS well penetrated through thus made GA film compared with a pure graphite film. Galvanostatic charge-discharge experiments were performed under the following conditions: cut-off voltage 3.2 V, current density 15 mA cm^−2^ using a cation exchange membrane (CEM) as a separator, where voltage was plotted against capacity (mAh). For testing the reliability of SSPAS, the charge and discharge measurements were repeated 10,000 times (Fig. 3a). As seen in Fig. 3a, except the first charging plot, the galvanostatic cycles are consistent within 5% deviations for 10,000 cycles. The average energy density was 0.45 Whkg^−1^ (based on the total mass of active materials) and 76% of the charge and discharge efficiency and the time spent for one cycle was 12 s. The detailed gas analysis was carried out after the charge-discharge measurements by the gas chromatogram using a Shimadzu GC-2014 under the helium gas flow at the rate of 25 ml min^−1^, where the whole cell was vacuumed before its opening for the analysis of inside gases. Hydrogen gas was not detected and oxygen was detected, but within the background. The results definitely indicate that SSPAS behaves well as the electrolyte at the operation voltage of 3.2 V.

The addition of activated carbon (AC) to both negative and positive electrodes forming a symmetrical capacitor increased the energy density remarkably as shown in Fig. 3b–e. Since the complete water free activated carbon was not easily obtained, the charge-discharge curves were affected by a small amount of water contained, the curves were unstable above 3 V. Therefore we chose the cut off-voltage at 3 V only in the capacitors consisting of the activated carbon mixtures. In Fig. 3b, cation exchange membrane (CEM) was used as the separator, while in Fig. 3c and d, a membrane filter (MF) and a filter paper (FP) were also used as separators, to compare with the result of using CEM. The result indicates that there exists little difference in the shapes of charge-discharge curves, and in the values of the energy density, that is 7.3~8.3 Whkg^−1^, by the use of these separators. The times spent for one cycle (Ts) were all similar in Fig. 3b,c and e to be about 300 s. The increased addition of AC increased the energy density to be 18.7 Whkg^−1^ at 40% AC (Fig. 3e).

### Effect of metal oxides for the graphite-based capacitor

It has been known that metal oxides contribute to store energy in capacitors. Among a variety metal oxides, we have examined the capability of SSPAS for graphite-based capacitors containing naturally abundant metal oxides such as Fe_2_O_3_ and Fe_3_O_4_, V_2_O_3_ and V_2_O_5_, and MnO_2_.

**Figure 4:**
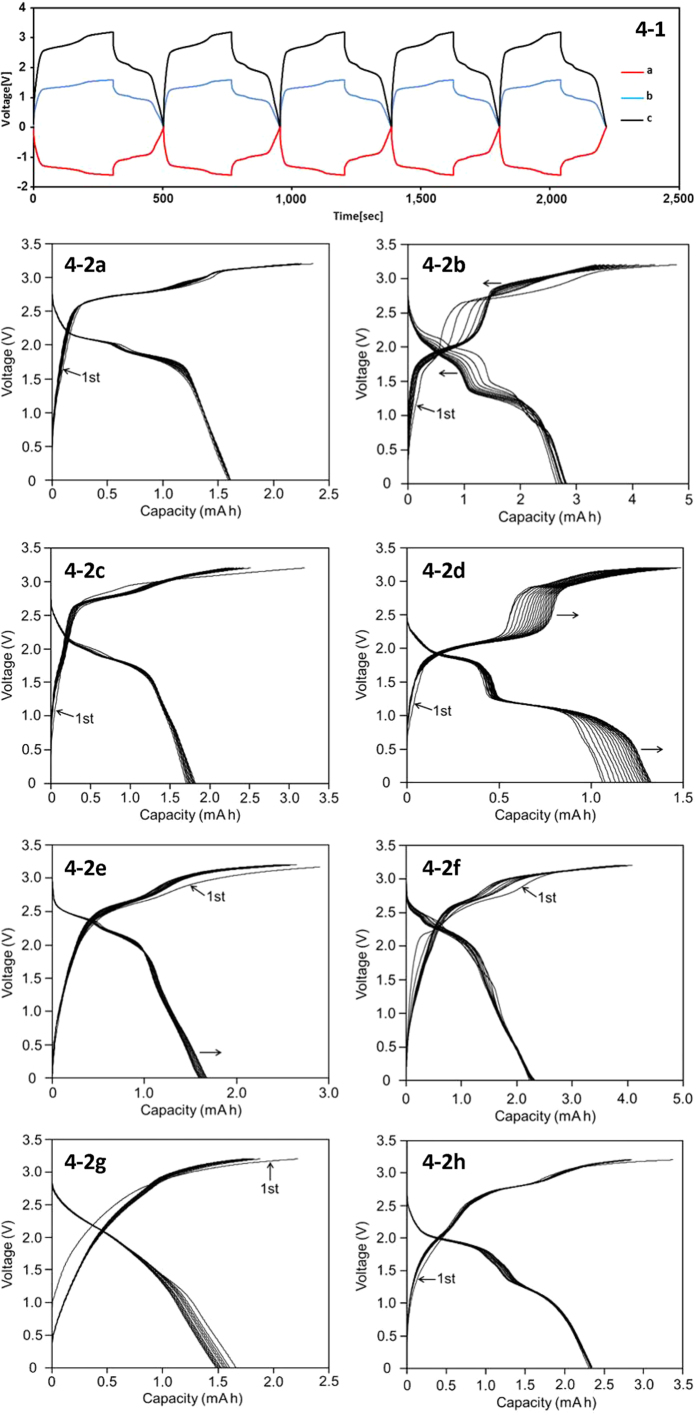
(1) Galvanostatic charge-discharge cycles plotted against time(s) by use of an Ag/AgCl reference electrode, where the electrode consisting of graphite mixture as a working electrode and the electrode containing 30% Fe_2_O_3_ as a counter electrode; (**a**) counter electrode, (**b**) working electrode, and (**c**) cell potential. (2) Charge-discharge cycles for various symmetric and asymmetric capacitors containing Fe_2_O_3_, Fe_3_O_4_, V_2_O_3_, V_2_O_5_ and MnO_2_. The capacitors are expressed as positive electrode/negative electrode. (**a**) GA/Fe_2_ O_3_ (30%), (**b**) Fe_2_O_3_ (30%)/Fe_2_O_3_ (30%). (**c**) GA/Fe_3_O_4_ (30%), (**d**) Fe_3_O_4_ (30%)/Fe_3_O_4_(30%), (**e**) GA/V_2_O_3_ (30%), (**f**) GA/V_2_O_5_ (30%), (**g**) MnO_2_(30%)/GA(20%AC), (**h**) MnO_2_(30%)/Fe_3_O_4_(30%).

First of all, we tried to examine the individual contributions in negative and positive electrodes especially for an asymmetric capacitor by using a reference Ag/AgCl electrode. We chose the electrode consisting of graphite mixture as a working electrode and the electrode containing 30% Fe_2_O_3_ as a counter electrode. Experiments were carried out under the N_2_ gas bubbling at 25 °C. The galvanostatic charge-discharge cycles plotted against time(s) are exhibited in Fig. 4-1, where (a) refers to the potential in the counter electrode, (b) the potential in the working electrode and (c) refers to the cell voltage. It can be seen in this figure that the plots (a) and (b) were almost symmetrical to the reference. This means that the electron absorption in the negative electrode and loss in the positive electrode takes place simultaneously. The cell voltage is the sum of the absolute values of (a) and (b).

In Fig. 4-2, the galvanostatic charge-discharge cycles, the cell voltages versus capacity (mAh), are shown for various symmetric and asymmetric capacitors containing metal oxides, Fe_2_O_3_, Fe_3_O_4_, V_2_O_3_, V_2_O_5_ and MnO_2_. As seen in these figures, the charge-discharge curves are different in shape from those of usual capacitors, particularly in the symmetric capacitors containing iron oxides, where the curves looked like those in typical rechargeable batteries having plateaus. The addition of metal oxides brought remarkable gain in the energy density. This may owe to the redox effects by metal oxides. It should be noted in symmetric capacitors (4-2b) and (4-2d) containing Fe_2_O_3_ and Fe_3_O_4_, respectively that the charge and discharge curves moved toward one direction according to the repetitions. It may be expected that a redox reaction takes place between Fe(II) and Fe(III). Similarly, redox reactions are also expected in the addition of V_2_O_3_ and V_2_O_5_, because vanadium possesses four oxidation states from V(II) to V(V). MnO_2_ is used in positive electrodes in (g) and (h) because of its electron emitting in nature. The results and specific conditions are summarized in Table 1.

**Figure 5:**
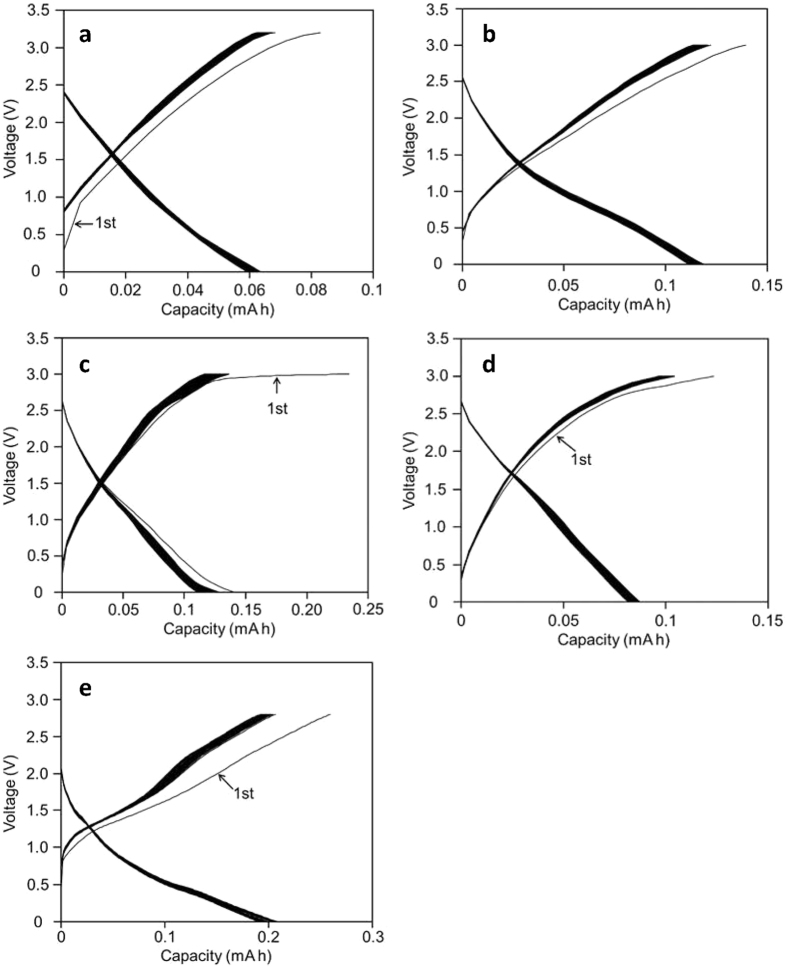
Galvanostatic charge-discharge cycles of the symmetric GA-based capacitors using saturated aqueous solutions of (**a**) LiClO_4_, (**b**) Mg(ClO_4_)_2_, (**c**) Ca(ClO_4_)_2_, (**d**) Ba(ClO_4_)_2_ and (**e**)Al(ClO_4_)_3_ as the electrolyte. Other specific conditions are described in Table 2. (**a**) LiClO_4_, (**b**) Mg(ClO_4_)_2_, (**c**) Ca(ClO_4_)_2_, (**d**) Ba(ClO_4_)_2_, (**e**) Al(ClO_4_)_3_.

Although sodium perchlorate is most soluble in any perchlorate salts, there exist other metal perchlorate salts of high solubility in water. Particularly, saturated lithium perchlorate aqueous solution behaves most similarly to SSPAS as exhibited in charge and discharge curves of a symmetric capacitor consisting of graphite mixture (Fig. 5a). However, our interest has been focused on more naturally abundant metal perchlorate salts, Mg(ClO_4_)_2_, Ca(ClO_4_)_2_, Ba(ClO_4_)_2_ and Al(ClO_4_)_3_. Galvanostatic cycles of symmetric capacitors containing these saturated aqueous solutions as electrolyte are shown in Fig. 5b–e. It is interesting to see in the discharge curves in (b) Mg(ClO_4_)_2_ and (e) Al(ClO_4_)_3_ that the curves are not linear, being larger in the capacity and hence having larger energy densities compared with SSPAS. This might owe to the partial reductions of Mg^2+^ and Al^3+^ to Mg and Al respectively during charging like as rechargeable batteries.

## Discussion

In Fig. 1, from the CVs of dilute aqueous solutions, the decomposition voltage shifts towards positive direction from basic (1 M NaOH) to acidic (1 M H_2_SO_4_) solutions as usual. The CV of SSPAS indicates that SSPAS is oxidized at more positive voltage than the decomposition voltage of 1MH_2_SO_4_ to be about +1.6 V and reduced at more negative voltage than the decomposition voltage of 1 M NaOH to be about −1.6 V. This leads to the potential window of SSPAS is approximately 3.2 V. The potential window thus determined above 3 V is largest being ever reported for any aqueous solutions. It should be noted that the CV curve for SSPAS is nearly symmetrical. Since the concentration of H_3_O^+^ and OH^−^ would be very low because of the lack of free water to hydrate in SSPAS, the electrolysis could be caused by the direct decomposition of the OH bond of water, and hence the potential barrier is expected to be the same in the oxidation and in the reduction. This reflects the nearly symmetrical CV curve of SSPAS.

The large potential window of SSPAS could be resulted from the weakened hydrogen bond of water. In order to evaluate the hydrogen bond, NMR measurements were performed, since the hydrogen bond of water is related to the chemical shift of ^1^H NMR signal. In an extreme case, under the supercritical condition, where water approaches to gaseous form and hence the hydrogen bond should be weakened, the chemical shift of ^1^H NMR signal of water moves to the higher magnetic field compared with those measured under normal conditions^20^. The ^1^H NMR of water was measured in SSPAS and in 1 M NaClO_4_ aqueous solution, respectively, and the chemical shifts were determined to be 3.69 ppm in SSPAS and 4.76 ppm in 1 M NaClO_4_ aqueous solution. This upfield shift in SSPAS is attributed to the weakened hydrogen bond in SSPAS despite the downfield contribution due to strong hydration towards Na^+^, though the quantitative contribution by the hydration is not known. Furthermore, the line-width at the half height in SSPAS was 0.0269 ppm, which was much smaller than 0.0483 ppm in 1 M NaClO_4_ aqueous solution. This narrowing of the line-width in SSPAS can be explained by the weakened dipole-dipole coupling between the neighboring proton spin through the hydrogen bond^21^.

The result in the CV measurement for SSPAS showing the large potential window was consistent with the stable galvanostatic charge-discharge performances at the high operation voltage in Fig. 3. In Fig. 3a, the galvanostatic charge-discharge cycles for the symmetric graphite-based capacitor were stable within 5% deviations for 10,000 cycles and the time spent for one cycle was 12 s. In this figure, the charging curves deviate from the linearity at the high applied voltage. We estimate in storing energy that the electrical double-layer process as illustrated in Fig. 2 could be combined by an additional redox process owing to an electron adsorption and an emission in the negative and positive electrodes, respectively as written bellow.


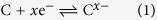



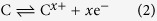


The deviations from the linearity in galvanostatic curves become larger in the presence of activated carbon (AC) as seen in Fig. 3b,c and d, where the discharge curves also deviate from the linearity. The energy densities increased more than the ten times by the addition of AC compared with the capacitor consisting of only graphite-acetylene black mixture (Fig. 3a). In Fig. 3b,c and d, the galvanostatic curves are similar with the similar energy densities in spite of using different separators, CEM, MF and FP, respectively. The time spent for one charge-discharge cycle (Ts) were about 300 s in all cases. These results indicate that the mobility of ions through separators is not important. As a matter of fact, Na^+^ and ClO_4_^−^ are able to pass through MF and FP, while only Na^+^ can pass through CEM. The increased addition of AC gives a considerable gain in the energy density as shown in Fig. 3e, i.e. the symmetric capacitor consisting of 40% activated carbon yielded the energy density of 18 Whkg^−1^. This value is quite large in carbon-based capacitors^14^,^21^,^22^, despite of using standard inexpensive carbon materials. It should be noted that the cycle time (Ts) also increased by the addition of AC from 12 s of the graphite-based capacitor to 750 s of the capacitor containing 40% AC. These results suggest that the storing energy does not proceed simply through an electric double-layer process, but proceeds through composite processes. On the assumption that the cycle time (Ts) is caused by the diffusion controlled mechanism for the simple graphite capacitor, the enlarged Ts due to the addition of AC would be attributed to a slower additional process. The latter slower process is estimated to be an electrochemical process such as an electron adsorption-emission reaction.

As exhibited in Fig. 4-2, the galvanostatic curves of the capacitors containing metal oxides are different in shapes from those of the carbon-based capacitors in Fig. 3. The discharge curves of the asymmetric capacitors containing Fe_2_O_3_, Fe_3_O_4_, V_2_O_3_ or V_2_O_5_ in the negative electrodes are all similar, i.e., slowly decrease until 1.7 V in the case of Fe_2_O_3_ and Fe_3_O_4_, and until 2.0 V in the case of V_2_O_3_ and V_2_O_5_, then decrease relatively fast to zero. Consequently, the energy density is larger in the later cases. As seen in Fig. 4-2b (containing Fe_2_O_3_) and Fig. 4-2d(containing Fe_3_O_4_), the charge and discharge curves of the symmetric capacity look no longer like capacitor, instead rechargeable batteries having plateaus at the voltage above 1.0 V.

A number of studies have be concerned with respect to the role of a variety of metal oxides, such as RuO_2_, MnO_2_, Fe_3_O_4_, IrO_2_ and V_2_O_5_ for capacitors^23^,^24^,^25^,^26^,^27^. Especially, RuO_2_ has been studied most extensively because of its conductivity and capability of fast reversible electron transfer between multiple oxidation states within 1.2 V as written below^15^.


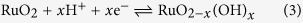


where 0 ≤ *x* ≤ 2

Since the acid equilibrium is unknown under the present condition in SSPAS, the equilibrium (3) might be preferably written as below.


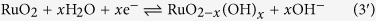


In the case of vanadium oxides V_2_O_n_ (where n is 3 or 5), the mechanism could be similar as written below.


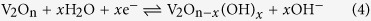


where x is not necessarily integer. The existence of equilibrium (4) was supported by the fact that the negative electrode became basic (pH = 11.3 measure after the dilution by water) after the full charge of capacitor. We do not deny the possibility of redox reactions of vanadium itself, because vanadium possesses four oxidation states from 2 to 5 within 1 V. On the other hand, in the positive electrode, the GA releases electron to form a positively charged form as described by the equation (2) or an equilibrium analogous to (4) as written below.


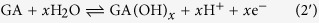


The equation (2′) would be more favorable than (2), because the positive electrode is acidic (pH = 2.31 measured after dilution by water) after the full charge.

**Figure 6:**
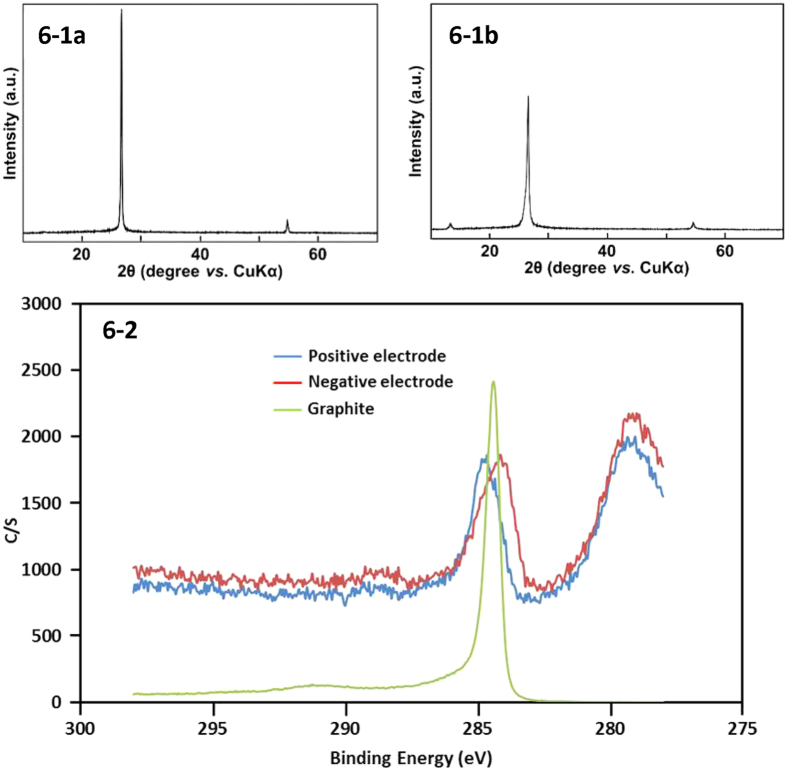
(1) XRD patterns in the negative (**a**) and positive (**b**) electrode for a capacitor consisting of 10% of V_2_O_3_ in the negative electrode and GA in the positive electrode. The samples were taken after the charge. (2) XPS spectra for carbon 1 s of graphite. The sample were taken in the same way as in Fig. 6(1) under the same condition. Because of the large 1 s signal of perchlorate oxygen, the S/N ratio of the sample signals are lower compared with the standard peak of original graphite (green). The red peak corresponds to the negative electrode and blue one positive electrode, respectively.

XRD measurements were carried out to examine the structural change in graphite mixture (GA) by the full charge of the capacitor, i.e., GA containing 10% V_2_O_3_ in the negative electrode and only GA in the positive electrode. As seen in Fig. 6-1, the diffraction pattern of the negative electrode exhibits a sharp peak at 2θ = 26°(a), which is characteristic of graphite^28^. On the other hand, the same peak at the positive electrode (b) is broadened after the charge. This indicates that the graphite in the positive electrode tends to lose a distinct structure to be amorphous according to the release of electron, but not to form graphite oxide^29^. A similar XRD result was also confirmed in the presence RuO_2_.

An XPS measurement was carried out for samples produced in the same way as described in Fig. 6-1 under the same condition by a ULVAC PHI 5000 VersaProbe III. The spectra are exhibited in Fig. 6-2 together with that of a graphite sample before use, which does not contain perchlorate. The peak corresponding to the graphite is assigned at 284.4 eV^30^ appearing at the middle. The spectra of the samples after use are observed at the edge of the large oxygen 1 s peak of perchlorate. The peak of the negative electrode (red line) shifts slightly to the lower energy and the peak of the positive electrode shifts slightly to the higher energy. The shifts are within 0.5 eV, which is too small to prospect any major change in 2p orbital of graphite during charge and discharge. As a matter of fact, peaks of graphite oxide were reported to be larger than 286 eV^30^. In conclusion, the result of XPS is in agreement with that of XRD, where major difference in chemical bond would not take place during charge and discharge.

As described above, the role of vanadium and iron oxides in the negative electrode is to adsorb electron during the charge. On the other hand, as seen in Fig. 4-2g, MnO_2_ was effective in the positive electrode. This means that MnO_2_ emits electron during the charge as reported in an earlier paper^5^. On the basis of the above assumption for the adsorption and for the emission of electron by metal oxides, we made capacitors containing 30% of MnO_2_ in the positive electrode. The charge and discharge cycles are exhibited in Fig. 4-2g and h, where GA with 20% AC and 30% Fe_3_O_4_ was involved in the negative electrode, respectively, and the energy densities and the time spent for one cycle (Ts) were 16.9 Whkg^−1^ and 24.1Whkg^−1^, 306 s and 442 s, respectively. The result indicates that Fe_3_O_4_ is more effective than AC for larger energy density in the capacitors containing MnO_2_ in the positive electrode. A capacitor containing 60% of Fe_3_O_4_ and MnO_2_ in the negative and in the positive electrode gave the energy density of 36.3 Whkg^−1^ and Ts was 700 s. The energy density is largest of the present series of experiments.

In earlier papers^5^,^7^,^14^, galvanostatic charge-discharge measurements for capacitors containing manganese oxide in positive electrodes were performed in aqueous solutions. The results are summarized as follows. **a**: MnO_2_/graphene in 1 M Na_2_SO_4_, operation voltage 2 V, current 5 mAcm^−2^ and Ts 900 s^5^, **b**: MnO_2_/AC in 2 M KNO_3_, operation voltage 2.2 V, current 100 mAg^−1^ and Ts 1200 s^7^, **c**: αMnO_2_/graphene in 6 M KOH, operation voltage 2 V, 1 Ag^−1^ and Ts 1000 s^14^. In these earlier studies, Ts was around 1000 s, though the conditions were all different. A quantitative comparison for these results involving the present study is difficult, since the materials used, the electrolytes and the galvanostatic conditions are all different. Even though, the charge-discharge rates are all similar from 700 s to 1200 s. This fact indicates that the rate of storing energy would be determined by electrochemical reactions instead of a diffusion controlled mechanism, and that SSPAS behaves well as equal as other dilute aqueous solutions.

It is interesting to see the charge and the discharge curbs for the symmetric capacitor containing Fe_2_O_3_ (Fig. 4-2b) and Fe_3_O_4_ (Fig. 4-2d), because they look like as rechargeable batteries having plateaus in the discharge curves, and furthermore the curves shift to one direction. We do not know in detail at moment, but it may be estimated that a redox reaction takes place between Fe(II) and Fe(III) during the charge and the discharge keeping the same oxide structures. The electron exchange reaction between Fe^2+^ and Fe^3+^ has been long known or it is the history of electron exchange reaction^31^. At moment, the detailed analysis is not possible, but it is important for understanding the charge-discharge mechanism in metal containing capacitors.

It has been found that perchlorate salts other than NaClO_4_, such as LiClO_4_, Mg(ClO_4_)_2_, Ca(ClO_4_)_2_, Ba(ClO_4_)_2_ and Al(ClO_4_)_3_, are very soluble in water and that their saturated aqueous solutions works as excellent electrolytes. Galvanostatic cycles of symmetric graphite-based capacitors containing saturated aqueous solutions of these salts are exhibited in Fig. 5 with their specific conditions in Table 2. The saturated lithium perchlorate aqueous solution is quite similar to SSPAS (Fig. 5a). However, we are more interested in naturally abundant other perchlorate salts. Particularly, the saturated Mg(ClO_4_)_2_ aqueous solution (SMPAS) is the superior electrolyte as well as SSPAS. As seen in Fig. 5b and Table 2, and the current density is 40 mAcm^−2^, which is largest at the present study, and hence raises the power density as large as 472 Wkg^−1^. The discharge curve deviates from a linear line being slow down at below 1 V and such a discharge curve increases the energy density to be 1.2 Whkg^−1^, which is more than double compared with 0.45 Whkg^−1^ in SSPAS. The time spent for one cycle is 10 s, which is shortest in the present study. A similar deviation from the linearity in the discharge curve was observed in the case of Al(ClO_4_)_3_. Since the standard redox potentials for Mg^2+^/Mg and Al^3+^/Al are −1.66 V and −2.36 V, respectively, the partial reductions of Mg^2+^ and Al^3+^ to metals could not be ruled out under the present conditions even though in consideration of strong hydrations toward Mg^2+^ and Al^3+^.

Recently, an aqueous electrolyte of using an eutectic hydrate melt which consists of a mixture of two organic lithium salts, that is Li(TFSI)_0.7_(BETI)_0.3_·2H_2_O, where TFSI is (bis(trifluoromethylsurphonyl)imide and BETI bis(pentauluoroethylsulphonyl)imide was reported for the use of Li ion battery^32^. The paper represents that this hydrate melt has a potential window over 3 V exhibiting an excellent capability for the Li ion battery. At moment, it is difficult to compare the difference in the above hydrate melt and saturated aqueous solutions of perchlorate salts as the electrolytes.

A need exists to use batteries or capacitors under extremely low temperatures. The eutectic temperature of SSPAS is −37 °C and that of SMPAS is even lower at −67 °C^13^. Considering these data, the capacitors of using SSPAS or SMPAS would be functionally operative at very low temperatures.

## Conclusion

Finally we conclude the present study. The most important finding was to successfully expand the potential window of the aqueous electrolyte first over 3 V by the use of the saturated sodium perchlorate aqueous solution (SSPAS). The electrolyte was demonstrated to behave well for graphite-based capacitors with respect to the stability for charge-discharge repetitions and the enlarged energy densities by the addition of activated carbon and metal oxides. It was also found that the other perchlorate electrolytes, particularly, the saturated Mg(ClO_4_)_2_ aqueous solution (SMPAS) is very feasible for the superior electrolyte. On the basis of the present results, together with the safety and economy impacts, SSPAS or SMPAS could replace non-aqueous electrolytes in commercial capacitors in the near future.

## Methods

A graphite capacitor was produced by the following procedure. A mixtures of carbon powder, i.e., graphite (J-SP-α of Nippon Graphite Industries Ltd.) 80%, acetylene black (Denka) 10%, carbon felt (TOYOBO) 10%, was pressed at 1 kgcm^−2^ by a hydraulic machinery to make a thin film. Thus made films were wetted by SSPAS and used for both negative and positive electrodes. These were assembled with a separator to make a capacitor using a Hohsen battery unit, where glassy carbons (Tokai carbon) are used in both current collectors. As separators, non-conductive water permeable sheets such as a cation exchange membrane (NEOSEPTA CIMS), a filter paper (ADVANTEC 5B) and a membrane filter (Millipore JVWP) and a PPS fiber (TORAY Torcon) were able to be used. Galvanostatic measurements were carried out by using an instrument of Bio-Logic VSP at temperature 30 °C. A Rigaku MiniFlexII was used for XRD measurements and a JNM-ECX400 P for NMR measurements.

### Safety test

It has been known that dried sodium perchlorate has potential danger of explosion in the presence of organic compounds. Therefore, we made a safety test as follows: the mixture of sodium perchlorate and graphite containing activated carbon or vanadium pentoxide was heated at 200 °C and examined the change of the mixture in increasing temperature. As a result, nothing was happened by the heating and we confirmed the safety of the capacitors.

## Additional Information

**How to cite this article**: Tomiyasu, H. *et al*. An aqueous electrolyte of the widest potential window and its superior capability for capacitors. *Sci. Rep.* **7**, 45048; doi: 10.1038/srep45048 (2017).

**Publisher's note:** Springer Nature remains neutral with regard to jurisdictional claims in published maps and institutional affiliations.

## Figures and Tables

**Table 1:** Performance of capacitors.

Figures	Operation Voltage (V)	Negative electrode	Positive electrode	Current density (mA cm^−2^)	Separator	Energy density (W h kg^−1^)	Power Density (W kg^−1^)
3a	3.2	GA	GA	15	CEM	0.45	205
3b	3.0	20% AC	20% AC	15	CEM	8.3	212
3c	3.0	20% AC	20% AC	15	MF	7.7	175
3d	3.0	20% AC	20% AC	15	FP	7.3	206
3e	3.0	40% AC	40% AC	10	CEM	18.7	200
4-2(a)	3.2	30% Fe_2_O_3_	GA	15	FP	18.3	340
4-2(b)	3.2	30% Fe_2_O_3_	30% Fe_2_O_3_	15	FP	28.7	327
4-2(c)	3.2	30% Fe_3_O_4_	GA	20	FP	19.6	336
4-2(d)	3.2	30% Fe_3_O_4_	30% Fe_3_O_4_	15	FP	11.2	276
4-2(e)	3.2	30% V_2_O_3_	GA	10	FP	20.1	198
4-2(f)	3.2	30% V_2_O_5_	GA	15	FP	27.8	252
4-2(g)	3.2	20% AC	30% MnO_2_	15	FP	16.9	303
4-2(h)	3.2	30% Fe_3_O_4_	30% MnO_2_	10	FP	24.1	350

**Table 2:** Specific conditions and results for the capacitors using saturated aqueous solutions of various metal perchlorate as electrolyte.

Electrolyte	Separator	Operation Voltage (V)	Current density (mA cm^−2^)	Energy density (W h kg^−1^)	Power density (W kg^−1^)
LiClO_4_	CEM	3.2	10	0.42	138
Mg(ClO_4_)_2_	CEM	3.0	40	1.2	472
Ca(ClO_4_)_2_	MF	3.0	15	0.85	200
Ba(ClO_4_)_2_	MF	3.0	15	0.64	209
Al(ClO_4_)_3_	FP	2.8	20	0.81	172

Graphite mixture (GA) was used in both negative and positive electrodes. CEM: Cation exchange membrane; MF: Membrane filter; FP: Paper filter.
